# Exploring the associations between the Self-structure of personality and problematic smartphone use in an adult sample

**DOI:** 10.1192/j.eurpsy.2023.2092

**Published:** 2023-07-19

**Authors:** S. Csibi, M. Csibi

**Affiliations:** 1 Sport Sciences; 2 Special Needs Education, Eszterházy Károly Catholic University, Eger, Hungary

## Abstract

**Introduction:**

Positive psychology theory sustains that the construct of the Self and its components, such as self-evaluation, social self-esteem, and self-coherence, determine our behavior. Personal daily habits and lifestyle modalities lay on these personality components. Problematic and addictive behavior is also strongly influenced by our Self and its main elements.

**Objectives:**

This study aims to determine those personality components related to the central Self-construct that actuates problematic smartphone use.

Our further objective is to identify targeted, self-enhancing activities that prevent problematic smartphone use.

**Methods:**

Participants were teenagers and adults (N=147) from the 17-73 age group (mean age 37.5 years), 31 male and 116 female.

Respondents provided self-reported data on their demographic characteristics, perceived self-esteem, social self-esteem, sense of coherence, and problematic smartphone use through an online survey attainable on a web-based platform.

Instruments were the Core Self-Evaluation Scale (Judge et al., 2003), the MOS-SSS Social Support Assessing Scale (Sherbourne & Stewart, 1991), the Sense of Coherence Scale (Rahe & Tolles, 2002), and the Smartphone Application-Based Addiction Scale (Csibi et al., 2018).

**Results:**

Respondents who reported being more familiar with smartphone applications and spending more time online scored higher on the problematic smartphone use scale. Our study found significant associations between age and problematic smartphone use, with those from younger groups scoring higher.

Participants characterized by lower self-esteem proved a more pronounced problematic smartphone use. In our sample, social self-image and social support did not show relevant correlations with the total score of problematic smartphone use.

However, a high sense of coherence showed a significant negative association with problematic smartphone use.

**Conclusions:**

A more mature Self-construct characterized by a positive self-evaluation and increased sense of coherence act as protective factors against problematic smartphone use.

Providing adequate self-evaluation and social support among young through targeted activities will have a higher role in younger age groups, preventing problematic smartphone use.

**Disclosure of Interest:**

None Declared

